# Exhaled breath condensate cysteinyl leukotrienes and airway remodeling in childhood asthma: a pilot study

**DOI:** 10.1186/1465-9921-7-63

**Published:** 2006-04-07

**Authors:** Christiane Lex, Angela Zacharasiewicz, Donald NR Payne, Nicola M Wilson, Andrew G Nicholson, Sergei A Kharitonov, Peter J Barnes, Andrew Bush

**Affiliations:** 1 Department of Paediatric Respiratory Medicine, Imperial College of Science, Technology and Medicine at the Royal Brompton Hospital and National Heart and Lung Institute, London, UK; 2 Department of Paediatric Cardiology and Pulmonology, Heinrich Heine University Duesseldorf, Duesseldorf, Germany; 3 Department of Paediatric and Adolescent Medicine, Pulmonary and Infectious Diseases, Wilhelminenspital Vienna, Austria; 4 Department of Histopathology, Imperial College of Science, Technology and Medicine at the Royal Brompton Hospital and National Heart and Lung Institute, London, UK; 5 Department of Thoracic Medicine, Imperial College of Science, Technology and Medicine at the Royal Brompton Hospital and National Heart and Lung Institute, London, UK

## Abstract

**Background:**

It has been suggested that cysteinyl leukotrienes (cysLTs) play an important role in airway remodeling. Previous reports have indicated that cysLTs augment human airway smooth muscle cell proliferation. Recently, cysLTs have been measured in exhaled breath condensate (EBC). The aim of this study was to evaluate the relationship between cysLTs in EBC and another marker of airway remodeling, reticular basement membrane (RBM) thickening, in endobronchial biopsies in children.

**Methods:**

29 children, aged 4–15 years, with moderate to severe persistent asthma, who underwent bronchoscopy as part of their clinical assessment, were included. Subjects underwent spirometry and EBC collection for cysLTs analysis, followed by bronchoscopy and endobronchial biopsy within 24 hours.

**Results:**

EBC cysLTs were significantly lower in asthmatic children who were treated with montelukast than in those who were not (median (interquartile range) 36.62 (22.60–101.05) versus 249.1 (74.21–526.36) pg/ml, p = 0.004). There was a significant relationship between EBC cysLTs and RBM thickness in the subgroup of children who were not treated with montelukast (n = 13, r = 0.75, p = 0.003).

**Conclusion:**

EBC cysLTs appear to be associated with RBM thickening in asthma.

## Background

Inflammation and remodeling are characteristics of the asthmatic airway. Until recently, remodeling was considered to be caused by longstanding inflammation, but this hypothesis has now begun to be questioned. Structural changes of remodeling have been found in childhood [1-3] and recently it has been shown that reticular basement membrane (RBM) thickening, a characteristic feature of remodeling, is present in children with difficult asthma to a similar extent to that seen in adults with asthma [3]. Studies in children are needed to explore the pathophysiology of airway remodeling in early life. However, bronchoscopy and endobronchial biopsy are not routine procedures in the management of childhood asthma. Therefore, non-invasive methods may help in studying airway remodeling in a similar way to those developed to investigate airway inflammation using exhaled nitric oxide, induced sputum or exhaled breath condensate (EBC).

Measurement of cysteinyl leukotrienes (cysLTs) in EBC could be one such potential non-invasive method of studying airway remodeling. CysLTs have been reported to play an important role in airway remodeling in asthma, with one study indicating that the cysLT_1_-receptor antagonist montelukast reduces airway smooth muscle cell hyperplasia and subepithelial fibrosis in ovalbumin-sensitised and -challenged mice [4]. In addition, cysLTs can be detected in EBC, with elevated levels reported in adults and children with asthma [5,6].

We measured EBC cysLTs in a group of children with moderate to severe persistent asthma undergoing bronchoscopy. We investigated whether cysLTs in EBC are lower in those patients taking regular montelukast, in addition to inhaled corticosteroids. In view of a potential role of cysLTs in airway remodeling, we examined the relationship between cysLTs in EBC and RBM thickness in endobronchial biopsies.

## Methods

### Patients

We recruited 29 children with moderate to severe persistent asthma who underwent bronchoscopy including endobronchial biopsy as part of their clinical assessment. Asthma was diagnosed according to American Thoracic Society (ATS) guidelines [7]. Moderate to severe persistent asthma was characterised by "daily symptoms despite >400 μg of inhaled budesonide (or equivalent)" [8]. 21/29 asthmatic children received additional treatment with systemic corticosteroids before bronchoscopy; of these 14 patients have been included into our difficult asthma protocol receiving a corticosteroid trial with either 2 weeks of prednisolone 40 mg/day (n = 8) or a single intramuscular injection of triamcinolone 80 mg (n = 6) before bronchoscopy [9]. Two patients had been prescribed systemic steroids for worsening of symptoms in the previous 2 weeks and 6 patients were using a long-term maintenance therapy with prednisolone up to 20 mg/day (table 1).

**Table 1:** Patients' characteristics

Number	29
Age (years)	10.2 (4–15)
Male, n	16
Atopic*, n	21
Duration of symptoms (years)	10.0 (5.0–12.0)
FEV_1 _(% predicted)#	80 (64–91)
Treatment in previous 2 weeks	
Inhaled steroid dosage (budesonide equivalent) (μg/day)	2000 (1000–2000)
Budesonide, n	7
Fluticasone, n	22
Oral and/or i.m. steroids, n	21
Long acting bronchodilator	28
Theophylline, n	1
Montelukast, n	14

Age data are expressed as mean (range). Other data are expressed as median (interquartile range) or absolute numbers (n).

*No data in 3 asthmatic children, ^# ^no data in five children who did not cooperate or could not withheld short-acting bronchodilator for 4 hours

### Study design

Subjects underwent spirometry and EBC collection, followed by bronchoscopy including endobronchial biopsy within 24 hours. Atopy was diagnosed if serum specific IgE (>0.34 kU/l) was raised or skin prick test was positive (wheal >2 mm larger than negative control) to at least one more antigen (*D. pteronyssinus*, cat, dog, grass pollen, *Aspergillus fumigatus*). Measurements of EBC and RBM thickness were done blind.

The study was approved by the Ethics Committee of the Royal Brompton and Harefield Hospital National Health Service Trust and written informed consent was obtained from all parents and children recruited into the study.

### Spirometry

Spirometry (Compact Vitalograph, Vitalograph Ltd, Buckingham, UK) was performed at baseline according to ATS guidelines [10]. Patients were asked to withhold short-acting bronchodilator for 4 hours prior to spirometry, if possible.

### Exhaled breath condensate

We collected EBC using a commercially available condenser (EcoScreen, Jaeger, Würzburg, Germany) according to the current ATS/ERS guidelines, as previously described [11,12]. Samples were immediately stored at -80°C. CysLTs (LTC_4_, LTD_4 _and LTE_4_) concentrations were analysed by a specific enzyme immunoassay (Cayman Chemical, Ann Arbor, MI). The lower limit for detection was 11.0 pg/ml and the upper limit was 700 pg/ml.

### Fiberoptic bronchoscopy and sample processing

Bronchoscopy with endobronchial biopsy was performed under general anesthesia as previously described [13]. Biopsies were taken from the subcarinae of the right lower lobe and processed into paraffin blocks. 5 μm sections were cut and stained with haematoxylin and eosin. Using light microscopy at × 400 magnification, coded sections were assessed for RBM thickness by a single observer (C.L.) using computer-aided image (NIH image 1.55, Maryland, US) as previously described [14]. 40 measurements were done at 20 μm intervals, RBM thickness was expressed as the geometric mean of the 40 measurements.

### Statistical analyses

All analyses were performed using the Statistical Package of the Social Sciences (SPSS). Comparison between groups was made using the Fisher's exact test and Mann Whitney U test as appropriate. Correlations were measured using Spearman rank correlation. P values < 0.05 were considered statistically significant.

## Results

Details of the subjects are shown in table 1. CysLTs in EBC were detectable in 24/29 asthmatic patients. Five subjects had values outside the limits of detection (3 were above, 2 below) and could therefore not be included into statistical analysis.

Suitable biopsies for the assessment of RBM thickness were obtained from 27 children. The median (interquartile range (IQR)) value was 8.78 (7.76–10.42) μm. There was no significant relationship between the inhaled steroid dose and RBM thickness (r = -0.25, p = 0.21) and there was no difference in RBM thickness whether patients received systemic steroids or not (p = 0.48).

### Analysis of asthmatic subgroups

**Figure 1:**
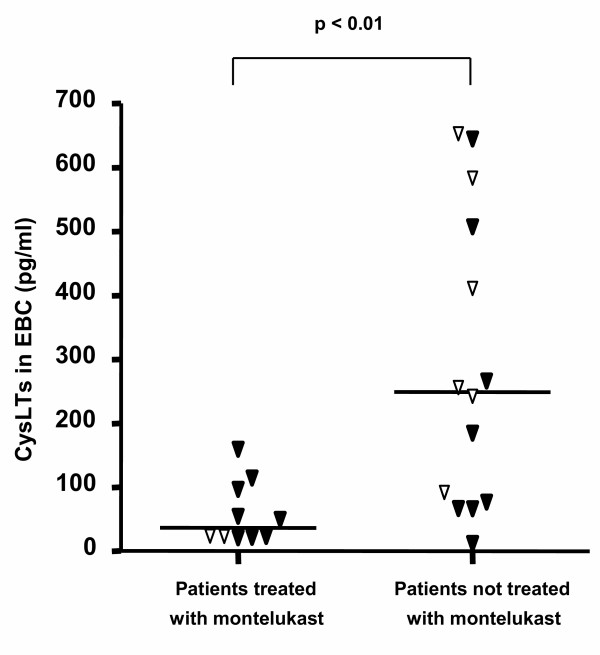
Relationship of exhaled cysteinyl leukotrienes (cysLTs) levels to montelukast therapy. Open symbols indicate patients without systemic steroids. Levels were significantly lower in asthmatic children treated with montelukast than in those who were not (36.62 (22.6–101.05) vs. 249.1 (74.21–526.36) pg/ml, p = 0.004).

**Figure 2:**
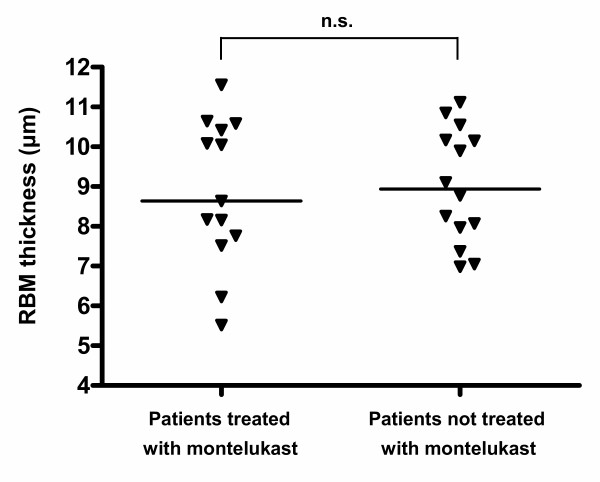
Relationship of reticular basement membrane (RBM) thickness to montelukast therapy. There was no significant difference between children treated with montelukast and those who were not.

Ten of 24 subjects from whom we obtained EBC values were taking montelukast (5–10 mg daily according to the patients' age). There was no significant difference in age, atopic status, inhaled steroid dosage, FEV_1 _or treatment with systemic steroids between patients who were and were not prescribed montelukast. CysLTs in EBC were significantly lower in asthmatic children treated with montelukast than in those who were not (median (IQR) 36.62 (22.60–101.05) vs. 249.1 (74.21–526.36) pg/ml, p = 0.004, figure 1). RBM thickness did not differ significantly between the two groups (p = 0.923, figure 2).

### Relationship between cysLTs in EBC and RBM thickness in endobronchial biopsies

**Figure 3:**
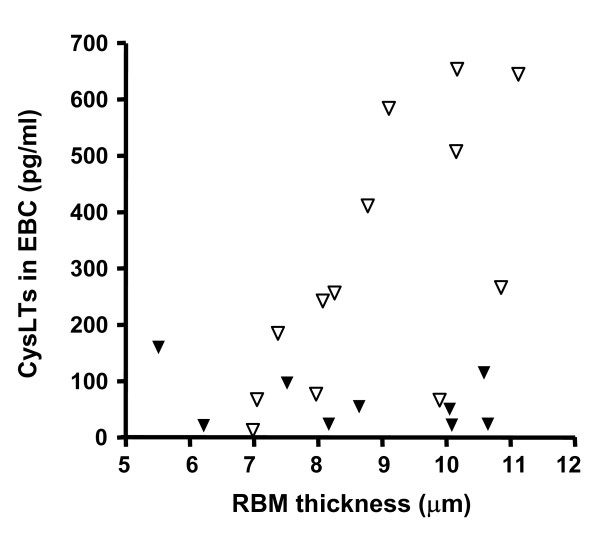
Relationship between exhaled cysteinyl leukotrienes (cysLTs) values and reticular basement membrane (RBM) thickness in endobronchial biopsies. Open symbols indicate patients without montelukast. There was a significant relationship between cysLTs levels in EBC and RBM thickness measured in endobronchial biopsies in the subgroup of patients who were not treated with montelukast (r = 0.75, p = 0.003).

Both suitable biopsies and measurable cysLTs levels in EBC were obtained in 22 children. There was a trend only for a significant relationship between cysLTs levels in EBC and RBM thickness measured in endobronchial biopsies in the group as a whole (r = 0.37, p = 0.09) (figure 3). However, there was a significant relationship between these two markers in a subgroup of children who were not additionally treated with montelukast (n = 13, r = 0.75, p = 0.003) (figure 3).

## Discussion

We have shown that exhaled cysLTs are detectable in children with moderate to persistent asthma with significantly lower concentrations in children who were additionally treated with montelukast compared to children who were not treated with montelukast. The principal finding of this study is that cysLTs in EBC correlated significantly with RBM thickness measured in endobronchial biopsies in a subgroup of asthmatic children who were not treated with montelukast. At the present time, we are not aware of any other study which has addressed these questions.

Csoma et al. have reported that cysLTs were detectable in all 13 children with moderate to severe persistent asthma who were included in their study, but no influence of montelukast was investigated since this drug was not given to any of the patients [6]. In our study, we have shown for the first time that the median exhaled cysLTs concentration was significantly lower in children who were treated with montelukast than in children who were not. In keeping with this Volovitz et al. have shown that montelukast treatment was associated with lower levels of cysLTs measured in nasal lavage of asthmatic children [15]. In an adult study Biernacki et al. found that exhaled cysLTs were significantly reduced after 4 weeks of treatment with montelukast [16]. Possible reasons for this are not clear, since this drug inhibits cys-LT_1 _receptors which should not affect the synthesis of cysLTs. However, recent evidence suggests that montelukast may have an inhibitory effect on cysLTs synthesis through an inhibitory effect on 5'-lipoxygenase, an essential enzyme in the biosynthesis of leukotrienes in addition to its antagonism of its receptors [17].

Several studies have indicated that cysLTs play a role in the pathophysiology of remodeling [18]. *In vitro *studies have shown that LTD_4 _augments epidermal growth factor-induced human airway smooth muscle proliferation [19] and that LTC_4 _upregulates collagenase expression and synthesis in human lung fibroblasts [20]. Furthermore animal models have shown that an increase in airway smooth muscle cells observed in allergen-treated Brown Norway rats was reduced by cysLT_1 _receptor antagonism [21]. In a recent mouse model Henderson et al. found that montelukast reduces airway smooth muscle cell hyperplasia and subepithelial fibrosis in ovalbumin-sensitised and -challenged mice [4]. In an open follow up study of seven asthmatic children low density areas at high resolution computerized tomography disappeared in six patients four years after additional treatment with montelukast suggesting that subepithelial fibrosis and air trapping may have been reduced by leukotrienes receptor antagonist treatment [22]. However, to our knowledge there are no human biopsy studies published so far supporting the role of cysLTs in airway remodeling.

In this study we have shown for the first time a possible relationship between cysLTs in EBC and an important feature of airway remodeling, namely RBM thickening, in asthmatic children. However, a significant correlation between cysLTs and RBM thickness was only present in subjects who were not treated with montelukast. This might be explained by the effects of montelukast on the cysLTs production, discussed above. The fact that treatment may perturb a relationship such as the one between cysLTs and RBM thickening is unsurprising. For example, the association between exhaled nitric oxide and eosinophilic airway inflammation measured directly is strongest in subjects not treated with anti-inflammatory treatment, or in asthmatics who are relatively insensitive to the effects of corticosteroids [9,23].

In order to study the relationship between cysLTs and RBM thickness and the use of exhaled cysLTs as a marker of airway remodeling further biopsy studies should be planned. Measurement of exhaled cysLTs and RBM thickness before and after a treatment with montelukast would be useful. However, the design of any study requiring repeat performance of bronchoscopy and endobronchial biopsy in order to obtain longitudinal data would be problematic.

There are a number of limitations to the current study. Firstly, only a relatively small number of a highly selected group of patients, those who underwent bronchoscopy for clinical indications, were tested, so the conclusions cannot be generalised to all children with asthma. The study should be regarded as a pilot study, hypothesis-generating, rather than definitive. Studies involving a larger sample size would be useful, although the numbers of subjects undergoing bronchoscopy and biopsy are always likely to be limited. Secondly, most patients were treated with high doses of inhaled and/or systemic steroids. Therefore, it is possible that cysLTs values in EBC would have been altered by the treatment. However, the influence of corticosteroids on cysteinyl leukotrienes is controversial. Dworski et al. found that treatment with oral prednisolone, while reducing asthma symptoms, had no effect on LTE_4 _concentration in bronchoalveolar lavage fluid [24]. Sebaldt et al. found that oral prednisolone had no effect on urinary LTE4 levels [25]. In contrast Baraldi et al. reported that cysLTs in EBC were reduced after a course of oral steroids given for an asthma exacerbation [26]. However, whether cysLTs in EBC are reduced after a steroid trial independently of an asthma exacerbation could not be studied. We acknowledge as well that prolonged high dose steroids may affect basement membrane thickness to a degree [27-29]. However, some studies do not support a role for corticosteroids in reducing airway remodeling [30,31]. However, again, the design of a study including patients who are not on steroids but undergo bronchoscopy would be problematic due to ethical considerations.

We acknowledge that our study is of cross-sectional observations, analysed by using coefficients of correlation. This limits the strength of the conclusions which can be drawn, and means that the study is hypothesis generating in many respects. Correlation analyses cannot prove either the presence or the direction of causality; this can only be done by appropriately designed, longitudinal studies. Furthermore, as a general point, statistically significant results may not necessarily be clinically relevant. Also, associations which are significant for groups may not be sufficiently sensitive to be used as a clinical tool for decision making in individual patients.

Another weakness is that we were unable to obtain data on the variability of repeated measurements of cysLTs and RBM thickness over time, due to ethical constraints for RBM thickness in particular. We have previously reported within-subject variability for RBM thickness in biopsies [32].

The cysLTs values in EBC were in general higher in our study in comparison to other studies [26,33]. This might be explained by the highly selected nature of the patients in our study. However, it has been noted that comparisons of data obtained in different laboratories are currently difficult because of the lack of standardised procedure for validated analytic techniques [11]. EBC analysis seems to be more reliable for making comparisons between groups, rather than for determining absolute levels of mediators [33].

## Conclusion

The goals of asthma therapy include prevention both of inflammation and structural airway wall changes. Whereas we have a number of non-invasive tools to measure inflammation (EBC, induced sputum, exhaled breath), non-invasive assessment monitoring of remodeling is much more difficult. This is the first study to investigate the relationship between cysLTs in EBC and one of the characteristic features of airway remodeling in asthma, RBM thickening. The data demonstrate an association between cysLTs and RBM thickening in those subjects not treated with montelukast. The use of measurements of cysLTs in EBC as a non-invasive marker of RBM thickening may be worth further investigation. If possible, future studies should include a larger number of children not treated with montelukast or systemic steroids. The design of such studies will require careful planning, in view of the ethical considerations. However, the validation of non-invasive methods of monitoring asthma in childhood will only be possible through the initial inclusion of invasive methods for comparison.

## Competing interests

AB has received funding from MSD, the manufacturers of montelukast for the following: attending academic meetings (European Respiratory Society, American Thoracic Society); for giving invited lectures; and unrestricted educational grants. He has no current financial relationship with the company.

## Authors' contributions

CL carried out the design of the study and the acquisition of data, performed the laboratory analysis, the quantification of the bronchial biopsies, the statistical analysis and interpretation of the data and drafted and revised the manuscript. AZ participated in the acquisition and analysis of data and the laboratory analysis. DNRP participated in the acquisition and analysis of the data and quantification of the bronchial biopsies and helped to draft the manuscript. NMW, AGN, SAK and PJB participated in the design and coordination of the study. AB conceived and coordinated the study, performed the bronchoscopies and obtained the endobronchial biopsies, and helped to interpret the data and to draft the manuscript. All authors read and approved the final manuscript.

**Table 2:** Corticosteroid therapy according to a treatment with montelukast

	Patients treated with Montelukast (n = 14)	Patients not treated with Montelukast (n = 15)
No systemic steroids	2	6
Prednisolone	7	9
• Maintenance therapy*	4	2
• 2 weeks course of prednisolone 40 mg/day	2	6
• Short course of prednisolone because of worsening of symptoms	1	1
Triamcinolone (one single i.m. injection)	5	1

* one child had triamcinolone and was on maintenance prednisolone therapy
